# Dual-Guided Semi-Supervised Semantic Segmentation for Citrus Quality Evaluation

**DOI:** 10.3390/foods15112029

**Published:** 2026-06-05

**Authors:** Xufeng Xu, Ruokai Guo, Kai Guo, Zetong Li, Zichao Wei, Xiuqin Rao

**Affiliations:** 1 College of Biosystems Engineering and Food Science, Zhejiang University, 866 Yuhangtang Road, Hangzhou 310058, China; 12213062@zju.edu.cn (X.X.); 22413018@zju.edu.cn (R.G.); 12413021@zju.edu.cn (K.G.); 22313051@zju.edu.cn (Z.L.); zichao_wei@zju.edu.cn (Z.W.); 2 State Key Laboratory of Agricultural Equipment Technology, Beijing 100083, China; 3 Zhejiang Key Laboratory of Intelligent Sensing and Robotics for Agriculture, Hangzhou 310058, China

**Keywords:** deep learning, semi-supervised learning, defect segmentation, pseudo-label, consistency regularization

## Abstract

Automated defect detection in precision agriculture serves as a critical technology for enhancing the quality of agricultural products. Although supervised-only semantic segmentation has demonstrated remarkable performance in citrus surface defect detection, it relies heavily on training with large-scale labeled data, which results in prohibitive acquisition costs. Semi-supervised learning mitigates reliance on labeled data by generating pseudo-labels. However, existing semi-supervised segmentation methods still face challenges. On the one hand, the instability of pseudo-labels and the propagation of noise can mislead the training of semi-supervised models. On the other hand, due to the lack of semantic constraints in feature learning, models often suffer from insufficient feature discriminability when handling complex samples, such as citrus surface defects characterized by similar textures and blurred boundaries. Therefore, this study proposes UP-ETS, a dual-guided semi-supervised semantic segmentation model based on the Mean Teacher–Student framework, specifically designed for the segmentation of complex citrus surface defects. UP-ETS employs Uncertainty Estimation (UE) based on Kullback–Leibler (KL) divergence to quantify the prediction discrepancy between the teacher and student models on blurred and ambiguous pixels. This mechanism guides the model to dynamically adjust weights, thereby reducing noise propagation and enhancing pseudo-label stability under complex citrus surface textures. Prototype Contrastive Learning (PCL) is utilized to align pixel-level features of difficult samples with class prototypes, optimizing the feature discriminability for complex citrus surfaces. Experimental results demonstrate that the UP-ETS model exhibits superior semi-supervised segmentation performance. Notably, at a labeled data ratio of only 1/16, the dice improved from 85.57% to 87.76% compared to the supervised-only baseline. Furthermore, the model shows significant performance enhancements in segmenting difficult samples, such as small targets, complex boundaries, and blurred regions. The results of ablation studies and t-SNE visualization prove the effectiveness of the proposed UE and PCL. These two methods synergistically guide the model to construct a feature space that is better structured and highly discriminative. Furthermore, UP-ETS outperforms various representative semi-supervised segmentation models in terms of segmentation performance, parameters, and inference speed. In cross-dataset validation, the model exhibits robust generalization capabilities, achieving performance comparable to supervised-only methods trained on the full augmented dataset. Consequently, the framework introduced in this study effectively mitigates the heavy dependency on annotated datasets, providing significant practical value for agricultural deployment.

## 1. Introduction

Citrus, characterized by its rich nutritional value, has become as one of the most significant economic crops cultivated and consumed worldwide [1,2]. With the rapid development of deep learning, semantic segmentation has gradually replaced traditional image processing techniques, emerging as the dominant method for detecting citrus surface defects [3]. It achieves pixel-wise classification by assigning labels to every pixel, offering comprehensive scene understanding and precise localization for better quality assessment [4].

Fully supervised deep learning models usually require substantial training data to perform well. While raw images are relatively easy to acquire, creating large-scale, high-quality semantic segmentation annotations is time-consuming and labor-intensive [5]. This challenge can be effectively addressed with semi-supervised learning. By utilizing limited labeled samples alongside abundant unlabeled data, it boosts both model performance and generalization. This method proves highly advantageous under conditions of limited ground-truth availability and prohibitive labeling expenses. Li et al. [6] proposed DM_CorrMatch, a semi-supervised semantic segmentation framework that utilizes innovative label updating and sample generation based on Denoising Diffusion Probabilistic Models (DDPM). This method successfully achieved the segmentation of rapeseed flower in Unmanned Aerial Vehicle (UAV) images, with an Intersection over Union (*IoU*) reaching 88.6%. Fan et al. [7] applied an improved semi-supervised method based on the Pyramid Scene Parsing Network (PSPNet) for apple leaf segmentation. Their results demonstrated that with labeled data ratios of 1/2, 1/4, and 1/8, the mean Intersection over Union (*mIoU*) reached 97.5%, 97.4%, and 96.5%, respectively, exhibiting performance comparable to supervised-only models. Casado-García et al. [8] evaluated the efficacy of three semi-supervised techniques for grape segmentation: pseudo-labeling, distillation, and model distillation. Experimental results indicated that all three methods improved segmentation performance, with average accuracy increasing by 5.62% to 6.01%. Notably, the model distillation approach yielded the most significant improvement. In summary, semi-supervised learning methods reduce the dependence on labeled data and enhance model performance by extracting additional information from abundant unlabeled data.

Semi-supervised semantic segmentation typically adopts a self-training paradigm. Specifically, an initial model predicts pseudo-labels for unlabeled data, which are subsequently merged with the labeled data for training [9]. Siddique et al. [10] employed a self-supervised learning strategy to automatically generate pseudo-labels from unlabeled data. By integrating data augmentation and semantic refinement, they achieved semi-supervised segmentation of fruit blossoms, outperforming state-of-the-art models on multiple fruit flower datasets. Furthermore, consistency regularization is another widely adopted strategy in semi-supervised semantic segmentation, typically implemented within a Teacher–Student framework. In this framework, the teacher model generates pseudo-labels from slightly altered unlabeled images. These pseudo-labels are then used to guide the student model’s predictions on significantly altered versions of the same images [11]. Consistency regularization methods aim to enforce prediction consistency across data, feature, and model levels, ensuring robustness against various perturbations. Methods such as MixMatch [12] and 3D-ViT [13] have integrated consistency regularization into pseudo-labeling strategies, achieving significant improvements on multiple datasets. For agricultural applications, in the task of Choy Sum segmentation, Yuan et al. [14] applied a semi-supervised model known as Adaptive Dynamic Mutual Training (AD-DMT). The AD-DMT framework employs a collaborative training strategy where a primary model generates pseudo-labels to guide a secondary network. To keep both models performing well together, this method adds an adaptive dynamic loss. Experimental evaluations demonstrate that the *mIoU* remains above 84.0% even when using different ratios of labeled data.

However, citrus surface defects are characterized by diverse shapes and complex features. The high visual similarity between defects and the background (healthy peel), coupled with ambiguous boundaries, poses significant challenges [15]. Such feature confusion often leads to a degradation in model segmentation accuracy. Due to the scarcity of labeled data, pseudo-labels generated by semi-supervised models during the early training stages are often unstable and contain noise. Particularly in ambiguous regions of citrus images, this noise tends to propagate during iterative training, leading to error accumulation and amplification, which ultimately degrades model performance. Recent studies have attempted to mitigate pseudo-label noise using cross pseudo-supervision (CPS), a method in which two networks provide each other with pseudo-labels for collaborative optimization [16]. Nevertheless, since CPS fundamentally relies on consistency regularization principles, the quality of pseudo-labels remains difficult to guarantee. Although confidence-based filtering can select reliable predictions for model training [17,18], determining an appropriate confidence threshold requires further investigation. A low threshold fails to effectively eliminate noise, whereas a high threshold may result in the loss of valuable information within the data. Moreover, the weak-to-strong consistency is a dominant strategy in semi-supervised learning. It enforces the model to predict consistent results on strongly augmented views using supervision signals from weakly augmented views [19]. Research indicates that adequate strong perturbations play a crucial role in boosting the model’s generalizability [20]. However, existing perturbations are primarily applied in the input, while consistency is constrained at the output. This may lead to a lack of semantic calibration for intermediate layer features. Consequently, when processing blurred regions on the citrus surface, the model is prone to generating wrong consistency guidance. Moreover, for regions with similar textures, relying solely on the consistency of output constraints is insufficient for discrimination at the feature level, resulting in the model’s inability to effectively distinguish subtle textural discrepancies.

To address the aforementioned challenges, this paper proposes UP-ETS, a semi-supervised semantic segmentation model for citrus surface defects based on a dual-guidance mechanism. Based on an EMA Teacher–Student framework that integrates pseudo-labeling and weak-to-strong consistency regularization, the model leverages a dual-guidance mechanism consisting of Uncertainty Estimation (UE) and Prototypical Contrastive Learning (PCL) to facilitate the semi-supervised learning process. This approach effectively extracts information from unlabeled data, thereby improving performance of defect segmentation. The main contributions of this study include:

(1) A pseudo-label optimization method based on UE is proposed. By utilizing KL divergence to quantify the uncertainty of pixel-wise predictions and dynamically adjusting loss weights of the model, this method addresses noise propagation in ambiguous regions, thereby enhancing the stability of pseudo-labels during early training stages.

(2) The PCL strategy is introduced to achieve pixel-level semantic feature alignment with class prototypes. This strategy enhances the feature discriminability of consistency regularization, effectively improving the model’s segmentation capability on difficult samples.

(3) The effectiveness and robustness of the proposed method are validated through ablation studies, comparative experiments, and cross-dataset generalization evaluations.

## 2. Materials and Methods

### 2.1. Image Acquisition and Dataset Creation

**Figure 1:**
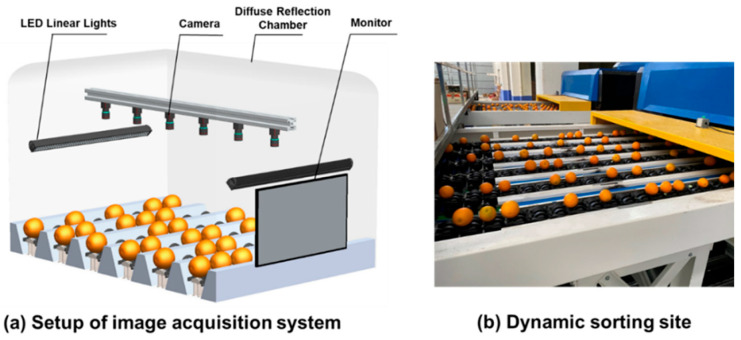
Overview of image acquisition system.

The images used in this study were primarily collected from a commercial orange sorting line in Yunnan, China, between 2022 and 2024 [4]. Figure 1 illustrates the online image acquisition system configuration, which integrates HIKROBOT cameras (MV-CS050-10GC-PRO, HIKROBOT, Hangzhou) and lens (MVL-MF0828M-8MP, HIKROBOT, Hangzhou) with a monitor. The setup also includes LED linear lights, a diffuse reflection chamber, and roller conveyors.

To maintain the details of defects, a cropping strategy rather than global downsampling was adopted during preprocessing. Consequently, 1719 images with a unified resolution of 224 × 224 pixels were generated for the dataset, preserving the original physical spatial resolution of the camera. The images were annotated using the LabelMe v6.3.1 (https://github.com/labelmeai/labelme, 16 December 2025), covering classes such as citrus, stem, and calyx, as well as three types of defects: canker, scarring, and deformity. With an 8:1:1 ratio, the images are partitioned into 1375 images for training, 171 for validation, and 172 for testing. Furthermore, the training set was subdivided into labeled and unlabeled subsets, with labeled ratios at 1/2, 1/4, 1/8, and 1/16, respectively. Detailed statistics of the dataset are presented in Table 1.

### 2.2. UP-ETS: Dual-Guided Semi-Supervised Semantic Segmentation Model

#### 2.2.1. Overview of the Proposed Methods

**Figure 2:**
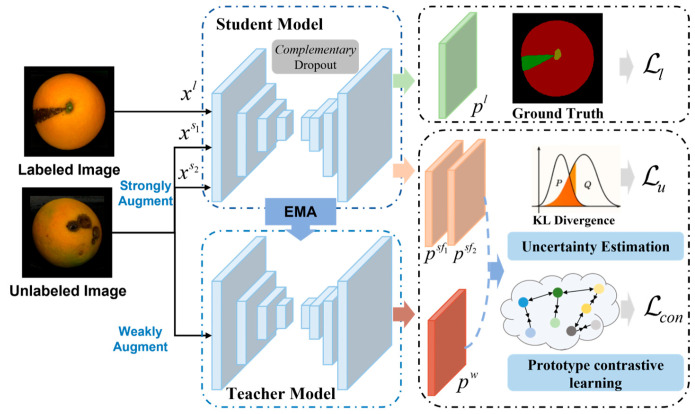
Framework of the dual-guided semi-supervised semantic segmentation model UP-ETS.

Figure 2 illustrates the framework of the proposed dual-guided semi-supervised semantic segmentation model. The model simultaneously inputs both labeled and unlabeled data. Specifically, unlabeled data undergo weak augmentation and two independent strong augmentations, respectively. The proposed model adopts the standard EMA Teacher–Student architecture. The teacher model generates pseudo-labels from weakly augmented images, while the student model processes strongly augmented images. By utilizing complementary Dropout to achieve unified augmentation at both image and feature levels, the student model is supervised by both the generated pseudo-labels and the labeled data. The teacher model is updated by the Exponential Moving Average (EMA) of the student model’s weights. To further enhance performance, UE and PCL are integrated to guide the optimization of pseudo-label quality and segmentation capabilities.

#### 2.2.2. Teacher–Student Model Based on Pseudo-Labeling and Weak-to-Strong Consistency Regularization

The Teacher–Student model is a widely adopted paradigm in semi-supervised semantic segmentation. It employs consistency regularization between the predictions of the teacher and student models on unlabeled data, enforcing the student model to produce consistent predictions under varying perturbations. Typically, the teacher model is updated as an EMA of the student model, thereby providing stable and reliable pseudo-labels to help the student network learn [21]. Both the EMA teacher and student models employ the Dense Prediction Transformer (DPT) architecture [22]. The DPT model adopts a Transformer-based encoder–decoder structure, in which the encoder utilizes the pretrained DINOv2.

The standard cross-entropy loss function is adopted as the supervised training objective for labeled images $p^{l}$ to minimize the discrepancy between the predictions pl and the ground truth labels $y^{l}$. This step is crucial for guaranteeing model convergence and providing initial guidance for pseudo-label generation. The supervised loss function is calculated as shown in Equation (1):

(1) $$L_{l}=L_{ce}(p^{l},y^{l})$$

where $L_{l}$ denotes the supervised loss function for labeled images, and $L_{ce}$ represents the standard cross-entropy loss function.

**Figure 3:**
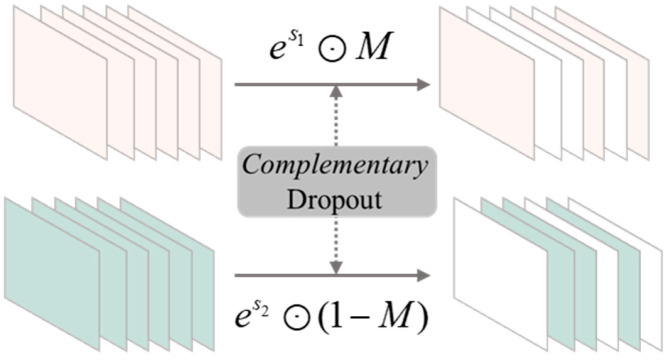
Schematic diagram of complementary Dropout mechanism.

For unlabeled images, weak augmentation and two independent strong augmentations are applied, respectively. Specifically, weak augmentation includes random scaling (range: 0.5 to 2.0), random cropping, and horizontal flipping. Strong augmentation comprises color jittering, grayscale conversion, gaussian blurring, and cutmix [23]. The weakly augmented image $x^{w}$ is input into the EMA teacher model to obtain prediction results and corresponding confidence scores. The result is only considered a valid pseudo-label $p^{w}$ when the prediction confidence exceeds a predefined threshold τ. Meanwhile, the strongly augmented images, $x^{s_{1}}$ and $x^{s_{2}}$, are fed into the student model. Here, the encoder simultaneously extracts two sets of features, which are mutually complemented in the feature dimension using the complementary Dropout mechanism. This achieves unified augmentation at both image and feature levels, thereby enhancing the model’s robustness and generalization ability [24]. The implementation principle of the complementary Dropout mechanism is illustrated in Figure 3.

Accordingly, the unsupervised loss function is given by:

(2) $$e^{s_{1}}\leftarrow e^{s_{1}}⊙M,\;e^{s_{2}}\leftarrow e^{s_{2}}⊙(1-M)$$

(3) $$p^{sf_{1}}=h(e^{s_{1}}),\;p^{sf_{2}}=h(e^{s_{2}})$$

(4) $${L^{\prime }}_{u}=\frac{1}{2}l[\max(p^{w})\geq \tau ](L_{ce}(p^{sf_{1}},{\hat{p}}^{w})+L_{ce}(p^{sf_{2}},{\hat{p}}^{w}))$$

where $l\left[\cdot \right]$ denotes the standard indicator function, which equals *l* if the condition inside is true (i.e., the maximum predicted probability exceeds the predefined threshold τ, and 0 otherwise. $e^{s_{1}}$ and $e^{s_{2}}$ denote the feature maps obtained by feeding the strongly augmented images into the student model’s encoder. *M* represents a randomly generated Dropout mask, specifically a binary mask sampled from a binomial distribution, where *M* and 1−M serve as complementary Dropout masks. $p^{sf_{1}}$ and $p^{sf_{2}}$ indicate the prediction results of the student model. h(·) denotes the output of the decoder. ${L^{\prime }}_{u}$ represents the unsupervised loss function for unlabeled data within the Teacher–Student framework.

In addition, the teacher model parameters are updated as an EMA of the student parameters, rather than being directly shared. This smoothing ensures the generation of stable pseudo-labels. The update equation is given by Equation (5).

(5) $$\theta^{t}\leftarrow \gamma \times \theta^{t}+(1-\gamma )\times \theta^{s}$$

where $\theta^{t}$ and $\theta^{s}$ stand for the weights of the teacher model and student model, respectively. γ acts as the smoothing factor.

#### 2.2.3. Pseudo-Label Optimization Guided by Uncertainty Estimation (UE)

**Figure 4:**
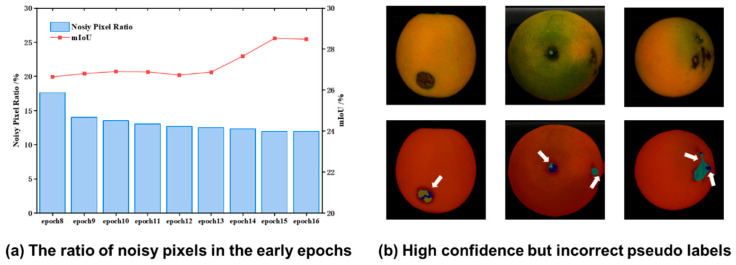
Noise pixel ratio and visualization of pseudo-labels in the initial training phase.

In the early training stages of semi-supervised models, pseudo-labels typically contain substantial noise. This noise hinders the ability to learn from unlabeled data and misleads model training, resulting in degraded performance. As illustrated in Figure 4a, the high noise level in pseudo-labels during the initial training phase directly impacts model performance. Figure 4b displays the pseudo-labels after applying high-confidence filtering. It is evident that wrong predictions persist even within these high-confidence regions. Therefore, it is essential to optimize the pseudo-labels to rectify incorrect predictions among the high-confidence samples.

To address the instability of pseudo-labels based on confidence thresholds, this study introduces UE to refine pseudo-label supervision. Specifically, the model selects predictions with high confidence and low uncertainty as pseudo-labels, which enhances their stability [25].

After obtaining predictions for the strongly and weakly augmented images, the Kullback–Leibler (KL) divergence is utilized to measure the information discrepancy between the output distributions. This process estimates uncertainty as an approximation of variance [26], as shown in Equation (6):

(6) $$\delta \left({\hat{p}}_{i}^{s},{\hat{p}}_{i}^{w}\right)={\hat{p}}_{i}^{w}\log\left(\frac{{\hat{p}}_{i}^{w}}{{\hat{p}}_{i}^{s}}\right)$$

where $\delta ({\hat{p}}_{i}^{s},{\hat{p}}_{i}^{w})$ denotes the KL divergence between the student and teacher predictions. ${\hat{p}}_{i}^{s}$ and ${\hat{p}}_{i}^{w}$ represent the softmax probability distributions output by the student and teacher models on the strongly and weakly augmented images, respectively.

A high KL divergence between the teacher and student models indicates that the generated pseudo-labels exhibit high uncertainty. These noisy pseudo-labels are mitigated by assigning adaptive weights based on estimated variance uncertainty. Additionally, a temperature scaling parameter *T_p_* is employed to optimize the softmax output, producing sharper pseudo-label distributions and minimizing class overlap. The specific definition is given in Equation (7):

(7) $$L_{ur}=\frac{1}{H\times W}\sum_{i=1}^{H\times W}\left[\omega_{i}L_{ce}\left({\hat{p}}_{i}^{s},\sigma \left(\frac{{\hat{p}}_{i}^{w}}{T_{p}}\right)\right)+\delta \left({\hat{p}}_{i}^{s},{\hat{p}}_{i}^{w}\right)\right]$$

where $i\in \left\{1,2,\ldots ,H\times W\right\}$ denotes the spatial index of the pixel across the height *H* and width *W* of the feature map. The dynamic weight variable $\omega_{i}=e^{-\delta \left({\hat{p}}_{i}^{s},{\hat{p}}_{i}^{w}\right)}$ is explicitly defined to adaptively adjust the supervision strength for each pixel based on its estimated uncertainty δ.

This strategy introduces uncertainty into the loss function to dynamically adjust pseudo-label weights. Consequently, it not only optimizes pseudo-label quality but also significantly improves the model’s robustness when learning from unlabeled data.

#### 2.2.4. Prototype-Guided Contrastive Learning (PCL)

**Figure 5:**
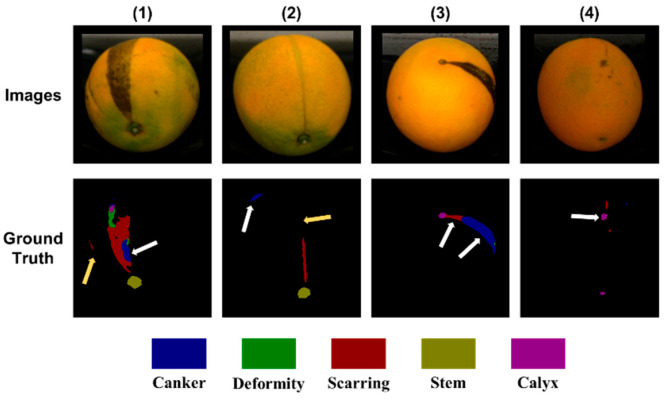
Error cases of semantic segmentation models in citrus surface defect segmentation. The yellow arrows indicate incorrect segmentation, while the white arrows indicate missed segmentation.

In citrus surface defect segmentation, the complexity and diversity of defects often lead to low segmentation accuracy, as illustrated in Figure 5. On the one hand, due to similarities in color, texture, or shape among certain defects, pixel misclassification is prone to occur if the segmentation model fails to learn discriminative features. Examples of such errors are indicated by the white arrows in Figure 5. On the other hand, some defects exhibit blurred boundaries. These areas are typically identified as low-confidence regions in the model’s predictions, leading to a reduction in their corresponding learning weights. Consequently, the model tends to neglect these boundary pixels, as indicated by the yellow arrows in Figure 5. Therefore, optimizing these difficult samples, characterized by ambiguity and blurred boundaries, is crucial for improving the performance of semi-supervised semantic segmentation models.

**Figure 6:**
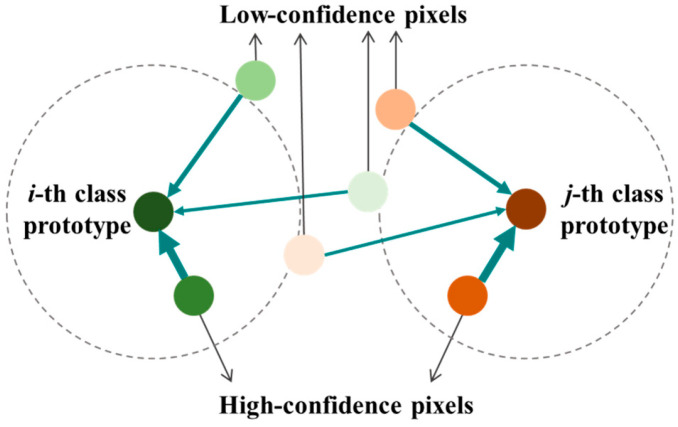
Schematic diagram of PCL.

Therefore, PCL is introduced to enhance the feature discrimination ability for these challenging samples. The objective of prototypical contrastive learning is to pull image features closer to their corresponding semantic prototypes, while pushing them away from the prototypes of different semantic categories. A prototype is defined as an aggregate representation of semantically similar samples, serving as a centroid of semantic category within the feature space. Integrating prototypes into contrastive learning imposes class-level semantic constraints on the feature space. By extracting and aggregating features from high-confidence samples to construct class prototypes, which serve as positive references, the features of low-confidence or unlabeled samples are pulled closer to their corresponding class centers under the guidance of contrastive loss [27]. The principle is illustrated in Figure 6.

Given the intermediate features $F_{s}\in {\mathbb{R}}^{B\times C\times H\times W}$ and the predicted semantic segmentation results $P_{s}\in {\mathbb{R}}^{B\times K\times H\times W}$ of the student model, where *B*, *C*, *H*, *W* denote the batch size, number of channels, height, and width of the feature maps, respectively. And *K* represents the number of classes. $P_{t}\in {\left\{0,1,\ldots ,K-1\right\}}^{H\times W}$ stands for the downsampled pseudo-labels created by the teacher model, while $M\in {\left\{0,1\right\}}^{H\times W}$ act as the downsampled confidence mask, respectively. M=1 indicates high-confidence pixels and M=0 represents low-confidence pixels. For each class *c*, the corresponding feature set collected from the high-confidence regions is defined as:

(8) $$F_{c}^{high}=\left\{f_{i}|P_{t}(i)=c,M(i)=1\right\}$$

Accordingly, the prototype of this class is defined as:

(9) $$P_{c}=\frac{1}{\left|F_{c}^{high}\right|}\sum_{f_{i}\in F_{c}^{high}}f_{i}$$

These prototypes, acting as high-confidence semantic centroid with robust stability and representativeness in the feature space, provide discriminative references for separating different categories.

For low-confidence pixels (M=0), their features $f_{i}^{low}$ and predicted classes are defined as follows:

(10) $${\hat{y}}_{i}=\mathrm{argmax}P_{s}(i)$$

These features are guided to be pulled closer to the positive prototype $p_{{\hat{y}}_{i}}$ and pushed away from the negative prototypes ${\left\{p_{j}\right\}}_{j\neq {\hat{y}}_{i}}$. Therefore, the PCL loss function is constructed as:

(11) Lcon=−1Nlogesimfilow,py^iτesimfilow,py^iτ+∑j≠y^iesimfilow,pjτ

where $sim(a,b)=\frac{a\cdot b}{\left\|a\right\|\left\|b\right\|}$ denotes the cosine similarity between two feature vectors. τ represents the temperature coefficient, used to regulate the smoothness of the similarity distribution; and *N* indicates the number of low-confidence samples.

To ensure computational viability on commercial-grade hardware, a feature-level spatial downsampling strategy is employed during the contrastive learning phase. Specifically, the pseudo-labels and confidence masks generated by the teacher model are spatially downsampled via interpolation to match the reduced resolution (H×W) of the intermediate features $F_{s}$. Performing contrastive learning within this compact feature space rather than at the original image resolution inherently bounds the maximum number of low-confidence samples *N* in Equation (11). This strategy prevents excessive memory overhead and potential out-of-memory issues associated with full-resolution, pixel-level contrastive computing.

Consequently, the loss function of the dual-guided semi-supervised semantic segmentation model is formulated as shown in Equation (12):

(12) $$L=L_{l}+L_{ur}+L_{con}$$

### 2.3. Evaluation Metrics of the Model Performance

In semantic segmentation tasks, model performance can be evaluated from multiple perspectives. Commonly used evaluation metrics include mean Pixel Accuracy (*mPA*), mean Intersection over Union (*mIoU*), Dice Coefficient (*Dice*), and Hausdorff Distance (*HD*). As detailed in Equation (13), *mPA* measures the share of accurate pixels among all pixels. *mIoU* means calculating the mean intersection and union results across each class, as shown in Equation (14). *Dice* measures the degree of overlap between the predicted and ground truth, as formulated in Equation (15). Finally, *HD* quantifies the distance between the two sets of labels and is conventionally utilized to assess boundary segmentation fidelity, as calculated in Equation (16).

(13) $$mPA=\frac{1}{k+1}\sum_{i=0}^{k}\frac{p_{ii}}{\sum_{j=0}^{k}p_{ij}}$$

(14) $$mIoU=\frac{1}{k+1}\sum_{i=0}^{k}\frac{p_{ii}}{\sum_{j=0}^{k}p_{ij}+\sum_{j=0}^{k}p_{ji}-p_{ii}}$$

(15) $$Dice=\frac{2}{k+1}\sum_{i=0}^{k}\frac{p_{ii}}{\sum_{j=0}^{k}p_{ij}+\sum_{j=0}^{k}p_{ji}}$$

(16) $$\left\{\begin{array}{c} HD(G,P)=\max\left[hd(G,P),hd(P,G)\right]\\ hd(G,P)=\underset{g\in G}{\max}\left\{\underset{p\in P}{\min}\left\|g-p\right\|\right\}\\ hd(P,G)=\underset{p\in P}{\max}\left\{\underset{g\in G}{\min}\left\|g-p\right\|\right\} \end{array}\right.$$

where $p_{ii}$ counts the pixels that truly belong to class *i* and predicted as class *i* correctly. $p_{ij}$ represents the class *i* pixels that the model wrongly predicted as class *j*. $p_{ji}$ indicates the number of pixels belonging to class *j* but predicted as class *i*. *k* stands for the total number of classes (excluding background). Regarding the *HD*, *G* and *P* denote the point sets representing the boundaries of the ground truth and predicted labels, respectively. The distance between them is defined as HD(G,P), also known as the bidirectional *HD*. hd(G,P) refers to the one-sided (or directed) *HD* from set *G* to *P*, which quantifies the maximum of the minimum distances from any point in *G* to all points in *P*. $\left\|g-p\right\|$ represents the Euclidean distance between two points.

### 2.4. Implementation Details

This study was conducted in a Linux environment (Ubuntu 20.04) using the PyTorch 1.11.0 framework and Python 3.8, with GPU acceleration enabled by CUDA 11.3. The hardware configuration consists of an Intel(R) Xeon(R) Gold 6147M CPU @ 2.50 GHz and four NVIDIA TITAN RTX GPUs (24 GB RAM each). To ensure optimal network convergence, the selected training hyperparameters are detailed in Table 2. The specific hyperparameter values listed in Table 2 were determined through preliminary grid search experiments to ensure stable model convergence and optimal segmentation performance.

Leveraging large-scale pre-training significantly reduces optimization time and provides robust prior feature representations. Because of this, the DPT network relies on the pre-trained DINOv2-small [28] to process image features.

## 3. Results and Discussion

### 3.1. Training Results of UP-ETS

**Figure 7:**
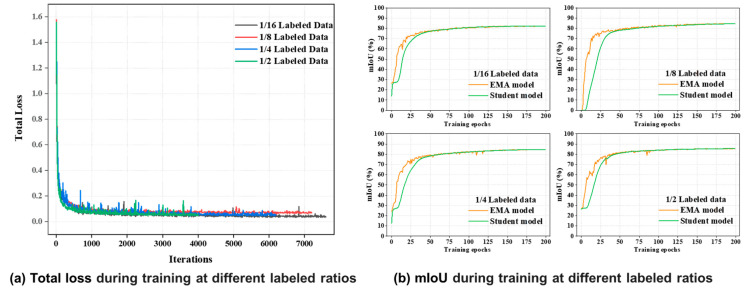
Training performance of the model under different labeled data ratios. (**a**) Total loss and (**b**) mIoU during training at different labeled data ratios.

Figure 7 illustrates the total loss, as well as the *mIoU* of both the student and teacher models, under different labeled data ratios. As observed in Figure 7a, the training loss goes down quickly at first and then stays flat for the 1/16 to 1/2 data ratios. Similarly, the *mIoU* of both the student and teacher models on the validation set exhibits a continuous increase and eventually stabilizes. This proves that no matter how much data is labeled, the model is capable of extracting effective information regarding the sample distribution from unlabeled data. Furthermore, the teacher model achieves a higher *mIoU* than the student model during the initial training stages. This capability allows the teacher model to generate high-quality pseudo-labels for the student model, improving the overall segmentation performance.

### 3.2. Comparison with Supervised Baseline

Table 3 presents a performance comparison between the proposed model and supervised-only baselines across different labeled data ratios. The baseline models were trained using supervised learning only, with no use of unlabeled data. The proposed model consistently outperformed the baselines in terms of mPA, mIoU, and Dice metrics across all labeled ratios. It is notable that the proposed model leads to greater performance improvements even at lower labeled ratios. Specifically, at ratios of 1/2, 1/4, 1/8, and 1/16, the mIoU improved by 1.67%, 2.47%, 2.69%, and 2.89%, respectively, compared to the baseline. By getting helpful information from unlabeled data, this approach easily solves the underfitting trouble found in standard supervised methods. By generating high-quality pseudo-labels for unlabeled data and integrating weak-to-strong consistency regularization, the model captures the underlying distribution patterns of the samples more effectively, thereby improving segmentation performance.

In terms of the boundary segmentation metric HD, UP-ETS outperformed the baseline when using 1/8, 1/4, and 1/2 of the labeled data, while performing slightly inferior to the baseline at the 1/16 ratio. Observing the IoU of every class, the model generally surpassed the baseline across different labeled ratios, particularly for scarring with complex boundaries and calyxes representing small targets.

**Figure 8:**
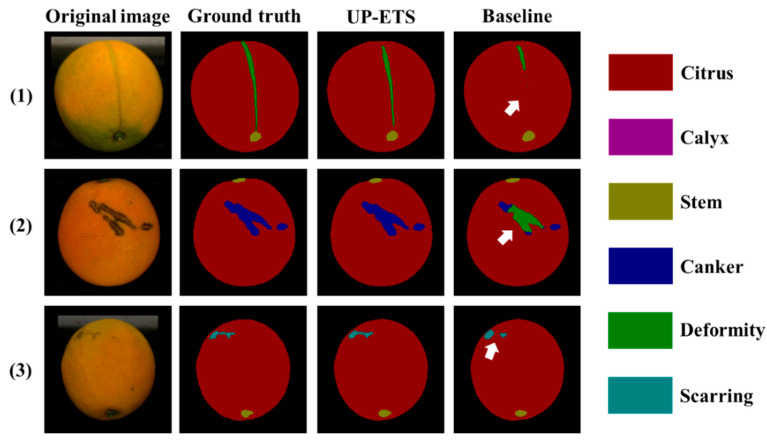
Segmentation outputs of the baseline and UP-ETS using a 1/8 data split.

As evidenced by the comparison of segmentation results at the 1/8 labeled ratio in Figure 8, UP-ETS demonstrated superior segmentation effects in the blurred regions [Figure 8 (1)] and complex boundaries [Figure 8 (3)]. Additionally, it achieved higher pixel classification accuracy than the baseline in the ambiguous regions [Figure 8 (2)]. These comparative results suggest that integrating PCL effectively improves the model’s ability to discriminate features, resulting in superior performance with small targets, complex boundaries, and blurred regions. By introducing class-level semantic constraints within the feature space, the model’s capability for fine-grained segmentation on difficult and unlabeled samples is strengthened, leads to an overall improvement in segmentation accuracy and robustness.

### 3.3. Ablation Studies

Ablation studies were conducted to validate the impact of the proposed improvements on segmentation performance.

To evaluate the impact of the temperature scaling parameter *T_p_* introduced in Equation (7) on the final probability distributions and overall model stability, a sensitivity analysis was conducted. Table 4 summarizes the segmentation performance across different *T_p_* values. In theory, a lower *T_p_* yields a sharper pseudo-label distribution. This minimizes class overlap and compels the model to make more confident predictions about pixels on ambiguous boundaries. As shown in Table 4, the model achieves an mIoU of 83.50% with *T_p_* = 0.9. However, decreasing the temperature further slightly degrades performance to 83.13%, as excessive sharpening can amplify confirmation bias by overweighting noisy signals. Conversely, a higher temperature results in softer distributions, which reduces the model’s discriminative ability. Based on this empirical evidence, *T_p_* = 0.9 is set as the default parameter to ensure optimal stability and pseudo-label quality.

Table 5 presents the results of mIoU at different labeled ratios in the ablation studies. As observed from the results, the UP-ETS achieved the best segmentation performance at all labeled ratios. In addition, the PCL and UE strategies that were introduced resulted in improvements compared to the baseline. Specifically, the mIoU increased by 2.67%, 2.85%, 2.88%, and 1.27% at labeled ratios of 1/16, 1/8, 1/4, and 1/2, respectively. This demonstrates that even in scenarios with scarce labeled data, the proposed improvements remain effective, consistently enhancing model performance.

**Figure 9:**
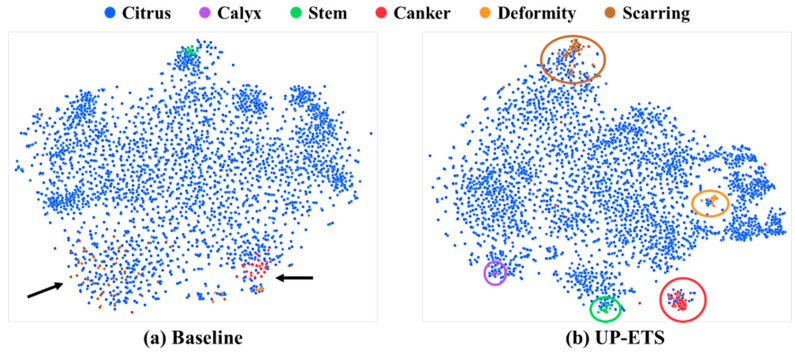
t-SNE visualization of feature distributions learned by UP-ETS and the baseline at 1/8 labeled ratio. The black arrows indicate pixels where the clustering results are poor.

To intuitively visualize the enhancement in the feature learning capability of the proposed model, this study employed t-SNE to perform dimensionality reduction on pixel-level features from the test set. Figure 9 illustrates the feature distributions learned by the baseline and UP-ETS at 1/8 labeled ratio. As observed from the feature distribution of the baseline model in Figure 9a, the features of the scarring are relatively dispersed. This lack of compactness poses challenges for dense pixel-wise prediction and the consistency learning required by semi-supervised models. Simultaneously, the features of deformity and canker exhibit overlap to some extent, with indistinct boundaries, making the model prone to confusion during prediction. In contrast, as illustrated in Figure 9b, the incorporation of PCL and UE effectively refines the feature space, resulting in more compact intra-class features and clearer boundaries between different categories. This demonstrates that PCL effectively aggregates intra-class features by constructing class prototypes, thereby enhancing feature discriminability. Furthermore, UE guides the model to focus on learning difficult samples by identifying regions of high uncertainty, facilitating the separation of ambiguous features.

In summary, the effectiveness of the proposed methods has been verified through ablation studies and t-SNE visualization. Both methods synergistically guide the model to construct a feature space that is better structured and highly discriminative, thereby enhancing the overall performance of the semi-supervised model.

### 3.4. Comparison with Other Representative Semi-Supervised Semantic Segmentation Models

Table 6 presents the *mIoU* results of UP-ETS compared with other representative semi-supervised semantic segmentation models at different labeled ratios. The representative models selected for comparison include AllSpark [29], FixMatch [19], and UniMatch [30]. In terms of segmentation performance, the proposed UP-ETS model outperforms other semi-supervised segmentation models in *mIoU* at all labeled data ratios.

**Figure 10:**
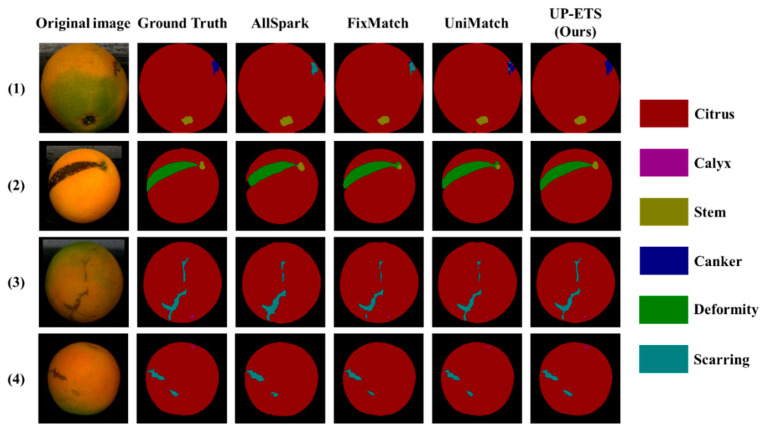
Comparison of segmentation results between UP-ETS and other representative segmentation models at a 1/8 labeled ratio.

As observed from the comparison of segmentation results in Figure 10, UP-ETS demonstrates superior segmentation effects compared to other models. It is evident from the results in Figure 10 (1) and (2), UP-ETS exhibits higher pixel classification accuracy. Even in regions with blurred semantic information (e.g., defects located at the edge of the field of view) and areas prone to confusion, UP-ETS maintains high classification accuracy, demonstrating strong feature discriminability. The segmentation targets in Figure 10 (3) and (4) correspond to scarring with irregular boundaries, which impose high demands on the model’s perception of fine-grained structures. In these cases, the segmentation masks generated by UP-ETS are significantly superior to those of other models in terms of boundary continuity and segmentation precision.

Defects on the surface of citrus fruits exhibit severe intra-class morphological variance and complex texture similarities with healthy peel. They also have highly ambiguous, transitional boundaries. Standard CNN implementations used for general defect detection usually rely on local receptive fields. These fields often have difficulty resolving these fuzzy biological boundaries, resulting in fragmented segmentation and misclassification of edge pixels. The proposed UP-ETS framework addresses these challenges with its specialized feature extraction mechanisms. PCL mechanism provides global semantic constraints, aggregating semantically similar features from the entire dataset into class prototypes. This pulls diverse, high-variance defect features closer to their respective semantic centers, significantly improving robustness against irregular textures like those of canker. UE module is highly sensitive to transitional zones between healthy and diseased tissues. The model mitigates the confirmation bias caused by ambiguous biological boundaries by dynamically weighting pseudo-labels based on variance.

Moreover, UP-ETS is a lightweight model with only 24.79 M parameters, fewer than the competing models. With an inference speed of 130.41 FPS, it fully satisfies the requirements for real-time defect segmentation.

### 3.5. Generalization on Other Datasets

To evaluate the generalization ability of the model, this study utilized the publicly available dataset Orange_Navel_1.5k (https://github.com/caixiongjiang/FastSegFormer, 16 December 2025) for testing. This dataset focuses on navel orange defects, including labels for wind scarring, ulcer, and sunburn [3]. It comprises a total of 1448 annotated images with a resolution of 512 × 512 pixels. The dataset was split following standard semi-supervised semantic segmentation protocols, as detailed in Table 7.

Table 8 presents the semi-supervised segmentation results of UP-ETS on the Orange_Navel_1.5k dataset. When using 1/16, 1/8, 1/4, and 1/2 of the labeled data, the UP-ETS model achieved *mIoU* scores of 87.46%, 84.31%, 88.29%, and 88.60%, respectively. This result demonstrates that the model exhibits excellent semi-supervised segmentation performance on this dataset as well. To further assess the performance of UP-ETS, this study compared it with FastSegFormer, a state-of-the-art fully supervised model proposed by Cai et al. FastSegFormer was trained on an augmented version of the Orange_Navel_1.5k dataset, comprising a total of 2604 training images. The results indicate that by leveraging unlabeled data, UP-ETS achieved performance comparable to that of FastSegFormer trained on the full augmented dataset, while using only 1/2 of the labeled samples.

In summary, the outstanding performance of UP-ETS in cross-dataset validation demonstrates the feasibility of leveraging unlabeled data within a semi-supervised framework. This study provides a valuable technical reference for addressing the challenge of labeled data scarcity in agricultural vision tasks.

## 4. Conclusions

To address the challenges of high annotation costs and low segmentation accuracy caused by complex defects in citrus surface defect detection, this study proposes UP-ETS, a dual-guided semi-supervised semantic segmentation model. By effectively enhancing the reliability of pseudo-labels through UE and strengthening feature discriminability using PCL, the model achieves accurate and robust segmentation of citrus surface defects. The segmentation performance of the UP-ETS model outperforms supervised-only baseline at all labeled data ratios, demonstrating significant improvements, particularly in low labeled ratios and the handling of difficult samples. Ablation studies and t-SNE visualization analysis confirm that the introduced UE and PCL function synergistically. By optimizing feature space representations, these strategies effectively enhance the segmentation capability for difficult samples, such as small targets and complex boundaries. Compared with various representative semi-supervised models, UP-ETS achieves superior segmentation accuracy while maintaining a lower parameter count and faster inference speed. Finally, the model demonstrates robust generalization capabilities when applied to other datasets. It achieves performance levels comparable to those of fully supervised models, even with limited labeled data.

In conclusion, the proposed UP-ETS enhances the performance of semantic segmentation models in semi-supervised tasks through a dual-guidance mechanism. It provides a data-efficient solution with superior performance and robust generalization potential for visual perception tasks in the field of precision agriculture.

While the proposed dual-guided semi-supervised framework demonstrates competitive performance and reduces annotation costs, this study has certain limitations. The primary dataset comprises 1719 images collected from a specific commercial sorting line under controlled lighting and background conditions. While these conditions reduce environmental variance and enable the model to focus on defect features, they may restrict the overall diversity of defects. In real-world agricultural applications, citrus surface defects can vary significantly across harvest seasons, regions, and varieties. Therefore, future work will focus on expanding the scale of the dataset and exploring cross-domain generalization techniques to enhance the robustness and adaptability in more diverse and complex industrial environments.

## Figures and Tables

**Table 1:** Details of dataset partition.

Dataset	Training Set				Validation Set	Test Set
Number of images	1375				171	172
Labeled ratio	1/2	1/4	1/8	1/16		
Number of labeled images	688	344	172	86		
Total	1719					

**Table 2:** Hyperparameter settings for model training.

Hyperparameters	Value
Batch Size	32
Epochs	200
Learning Rate	0.000005
Initial Learning Rate	0.00002
Learning Rate Scheduler	Poly Scheduler $lr\leftarrow lr\times {\left(1-\frac{iter}{\mathrm{total\_iter}}\right)}^{0.9}$
Optimizer	AdamW
Weight Decay	0.01

**Table 3:** Performance comparison between UP-ETS and supervised-only baseline under different labeled data ratios.

Labeled Ratio		1/16		1/8		1/4		1/2	
Model		UP-ETS	Baseline	UP-ETS	Baseline	UP-ETS	Baseline	UP-ETS	Baseline
*mPA* (%)		89.60	87.74	90.14	89.42	90.44	89.35	91.64	89.83
*mIoU* (%)		82.97	80.64	83.50	81.31	84.67	82.63	85.28	83.88
*Dice* (%)		87.76	85.57	88.33	86.13	88.96	87.62	89.81	88.85
*HD*		6.29	6.23	5.69	6.80	5.12	6.03	4.90	5.44
*IoU* (%)	citrus	98.34	98.19	98.29	98.31	98.39	98.27	98.44	98.33
	calyx	66.55	56.66	64.10	61.67	67.40	61.98	70.51	63.07
	stem	78.47	73.98	77.34	75.92	79.03	76.25	78.99	77.14
	deformity	72.97	76.04	83.61	78.42	85.35	82.27	86.20	82.97
	canker	83.84	79.85	84.47	80.16	85.04	84.65	86.48	84.17
	scarring	77.40	74.96	77.74	75.04	78.57	76.12	79.70	76.59

**Table 4:** Segmentation performance across different *T_p_* values at labeled ratio of 1/8.

*T_p_*	0.8	0.9	1.0	1.1	1.2
*mIoU* (%)	83.13	83.50	82.86	83.30	82.24

**Table 5:** Ablation study on PCL and UE.

Models	*mIoU* (%)			
	1/16	1/8	1/4	1/2
Baseline	80.81	81.19	82.30	84.21
Baseline + PCL	82.67	83.32	84.36	85.13
Baseline + UE	82.21	83.40	84.80	84.98
Baseline + PCL + UE UP-ETS (Ours)	82.97	83.50	84.67	85.28

**Table 6:** Performance comparison between UP-ETS and other representative semi-supervised models.

Models	*mIoU* (%)				Parameters (M)	Inference Speed ^1^ (FPS)
	1/16	1/8	1/4	1/2		
AllSpark	62.45	63.28	64.48	65.28	32.07	71.45
FixMatch	79.88	80.13	80.19	81.01	59.46	91.66
Unimatch	80.04	80.65	80.72	82.23	59.46	93.83
UP-ETS (Ours)	82.97	83.50	84.67	85.28	24.79	130.41

^1^ The inference speed was tested on a single NVIDIA RTX 3060 GPU.

**Table 7:** Details of Orange_Navel_1.5k dataset partition.

Dataset	Training Set				Validation Set	Test Set
Number of images	1158				144	145
Labeled ratio	1/2	1/4	1/8	1/16		
Number of labeled images	579	290	145	73		
Total	1448					

**Table 8:** Semi-supervised segmentation results of UP-ETS on the Orange_Navel_1.5k dataset.

		Labeled Ratio				FastSegFormer-P † ED [3]
		1/16	1/8	1/4	1/2	
*mIoU* (%)		87.46	88.29	88.50	88.60	88.57
*IoU* (%)	sunburn	86.61	88.74	89.78	87.91	89.34
	ulcer	88.33	88.31	89.00	89.52	87.50
	wind scarring	75.54	76.70	75.81	77.55	78.09

†: Backbone network was pretrained in ImageNet-1K. ED: Enhanced datasets.

## Data Availability

The original contributions presented in the study are included in the article, further inquiries can be directed to the corresponding author.
